# Differential Gene Expression Regulated by Oscillatory Transcription Factors

**DOI:** 10.1371/journal.pone.0030283

**Published:** 2012-01-24

**Authors:** Luca Cerone, Zoltán Neufeld

**Affiliations:** 1 School of Mathematical Sciences and Complex and Adaptive Systems Laboratory, University College Dublin, Dublin, Ireland; 2 School of Mathematics and Physics, University of Queensland, Brisbane, Australia; Inserm U869, France

## Abstract

Cells respond to changes in the internal and external environment by a complex regulatory system whose end-point is the activation of transcription factors controlling the expression of a pool of ad-hoc genes. Recent experiments have shown that certain stimuli may trigger oscillations in the concentration of transcription factors such as NF-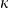
B and p53 influencing the final outcome of the genetic response. In this study we investigate the role of oscillations in the case of three different well known gene regulatory mechanisms using mathematical models based on ordinary differential equations and numerical simulations. We considered the cases of direct regulation, two-step regulation and feed-forward loops, and characterized their response to oscillatory input signals both analytically and numerically. We show that in the case of indirect two-step regulation the expression of genes can be turned on or off in a frequency dependent manner, and that feed-forward loops are also able to selectively respond to the temporal profile of oscillating transcription factors.

## Introduction

Cells are dynamic environments constantly adapting themselves to internal and external stimuli. The response to such stimuli is a tightly controlled multi-step process from sensing the stimulus, usually by means of receptors present in the external and internal membrane, transmission of the signal across the cell by a cascade of protein modifications and protein-protein interactions, that activates specific transcription factors which, in turn, regulate the expression of target genes. Fine tuning regulations, e.g. post-translational and post-transcriptional modifications, take place at every step in process providing robustness against noise, specificity to the triggering stimulus and insulation between the different pathways.

Recent discoveries have revealed that transcriptional regulation itself is a very complex process and genes are not just activated or deactivated by transcription factors. Rather transcription factors activate a pool of genes [1] that share a high level of connectivity forming transcriptional networks in which the expression of one gene controls in turn the expression of others generating temporal expression programs. Determining the dynamics of the genetic response from the topology of transcriptional networks is not always straightforward therefore it is important to develop new theoretical and experimental approaches to better understand the mechanisms responsible for regulating gene expression.

Some insights have been gained from identifying so called *network motifs*. Network motifs are patterns of connectivity that are present in a much higher frequency than in a network of similar dimensions but whose links between its nodes are generated randomly [1]. As the network motifs recur in different organisms, and have been selected by evolution over other possible configurations, they are thought to have special relevance in biological systems, and certain features linked to their topology have been identified [2], [3]. For example, negative auto-regulation, occurring when a gene promotes its own inhibition, has been shown both theoretically and experimentally to be used by cells to speed up the response of gene expression and to promote robustness to fluctuations in production rates [4]. On the other hand positive auto-regulation slows down the response [2], and can lead to bistability [5]–[7] keeping the gene active or inactive even after the stimulus is turned off. The role of certain network motifs in selectively responding to signals depending on their temporal structure has also been studied [8].

Among the network motifs feed-forward loops have been widely investigated both theoretically and experimentally and many of their properties have been described, such as persistence detection, protecting against transient loss of signals [9], generating pulses of expression [10], e.g. playing a role in the temporal organization of the cell cycle [11], speeding up the response [12], detecting fold over basal expression [13], [14], or generating non monotonic response functions [15]. In most previous studies the response of the target genes was studied in the case of a persistent step-like on/off stimulus. However it is becoming more and more evident that more complex temporal patterns in protein concentrations and sequential activation by oscillatory signals can play an important role in determining the outcome of gene expression.

Oscillations have been observed for a long time in the most varied biological systems e.g. cell cycle [11], neuronal firing, heart beat arising as an emergent property of thousands of cells, embryogenesis [16], calcium oscillations associated with differential activation of transcription factors [17], frequency modulated calcium dependent gene expression of Crz1 [18], [19], and in the concentration of transcription factors such as p53 [20], [21], HES-1 [22] and NF-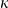
B [23]–[25].

For transcription factors the functional role of oscillations is not well understood. A number of studies provide supporting evidence that the oscillatory temporal dynamics of nuclear NF-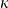
B may encode information about the required genetic response [25]–[27]. Moreover it has recently been shown that for cells stimulated by TNF-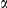, oscillations in the dynamics of gene expression are a widespread phenomenon [28], [29] occurring in almost 15% of the human genome. These oscillations occur not only in genes targeted by NF-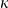
B, suggesting that other oscillatory transcription factors may exist and that the oscillations may propagate to other pathways through the transcriptional regulatory network, for example TNF-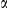 stimulated cells also show oscillations induced in MAP kinase activity [30].

In this work we theoretically and numerically investigate how the transcriptional activity of genes regulated by simple network motifs is affected by oscillations in the concentration of transcription factors. First we study and characterize quantitatively the properties of direct regulation. We then use and extend these results to understand the behavior of indirect two-step regulation and feed-forward loops, driven by oscillating transcription factors with varying period and temporal profile. The specific aim is to analyze how various characteristics of the oscillatory input signal (e.g. frequency and shape) can control differential expression of genes, that is not possible in the case of steady state responses. A better understanding of such mechanisms based on theoretical models can help identifying the functional role of experimentally observed oscillations in the expression of various genes. We focus on the genetic response produced by synthetic oscillatory input signals, where we can directly control the different characteristics of the signal.

## Methods

**Figure 1:**
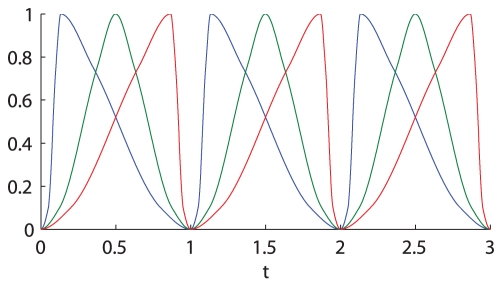
Constuction of the input signal 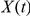 with different shapes. The plot shows the 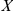 signal skewed to left (blue), the symmetric one (green) and the one skewed to right (red), with a period of 1 h. The shape of the signals have been chosen so that all of the three signals are above the value 0.1 for 75% of the time and above the value 0.75 for 25% of the time. The shape of the blue curve has been obtained interpolating the points (0,0) (0.05, 0.1) (0.1,0.75) (0.1333,1.0000) (0.35,0.75) (0.8, 0.1) (1, 0); the green curve interpolating the points (0,0) (0.125,0.1) (0.375, 0.75) (0.5,1) (0.625, 0.75) (0.875, 0.1) (1, 0); the red curve interpolating the points (0, 0) (0.2, 0.1) (0.65, 0.75) (0.8667, 1) (0.9, 0.75) (0.95, 0.1) (1,0).

In the following we present and analyze differential equation based models that link the temporal dynamics of a transcription factor 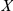 to the expression of the targeted genes. We investigate the effects of changing the oscillation period and the shape of the temporal profile of the concentration 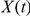 while its average value remains the same. We assume that the concentration of the transcription factor is normalized so that 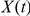 varies between 0 and 1. We choose the temporal profiles of the input signal 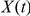 such that it is above the value 0.1 for 75% of the time and above the value 0.75 for 25% of the time. We have considered the three cases shown in Figure 1, in which 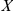 builds up rapidly and decreases slowly (blue curve), the *symmetric* case in which 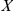 goes up and down in the same amount of time (green curve) and the case in which 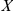 increases slowly and decreases quickly (red curve). The temporal profiles have been obtained by spline interpolation across the selected points over the time interval [0, 1] (see caption of Figure 1), and then stretching and repeating them so that 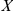 produces a periodically oscillating signal.

Analytical solutions for the components of the considered mechanisms have been derived (see Results section) and have been used to run the simulations presented in this work.

## Results

### Direct gene regulation

We first studied the effects of oscillations on the average expression of a gene 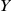 when its transcription is directly regulated by the transcription factor 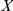. We assume that 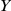 is synthesized at a rate 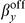 when the concentration of 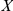 is below a certain threshold 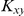 and at a rate 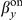 when 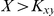. Thus, the analog signal 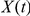 is converted into the digital signal 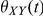 that is 1 when 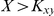 and 0 otherwise. If 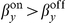 then 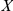 is an activator for the gene Y, else it is an inhibitor. For the sake of clarity in the following we assume that 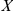 is an activator, but analogous results can be obtained for inhibitors. We assume that 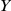 is degraded following mass action kinetics with decay rate 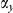. Thus the expression of 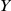 can be described by the differential equation
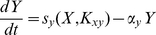
(1)where 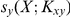 is the step function
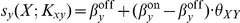
A similar formulation of the model could be given by assuming a Hill rate function for the up-regulation of the synthesis of 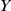 by 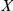 as:
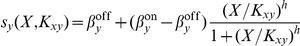
that becomes equivalent to step-function above in the limit when 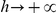. We will use the form with the step function as a simple approximation for the gene activation, since that somewhat simplifies the analysis of the models and can help understanding of the basic mechanisms governing gene responses [2]. Although this simplification may slightly modify the dynamics of the expression level of 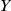, the qualitative behavior remains the same (see Supporting Information S1).

**Figure 2:**
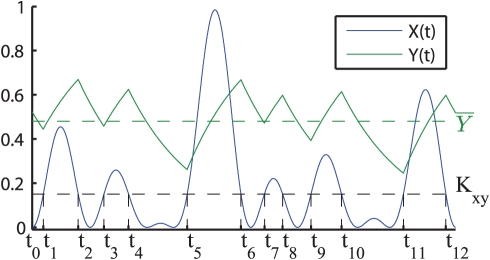
Example of direct regulation. The Figure shows the temporal dynamics of a transcription factor 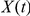 (blue), and the response of the directly regulated gene 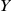 (green) at stationary regime. As 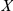 increases and decreases it crosses the threshold of activation 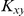 (dashed line) determining a sequence of intervals 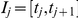 such that 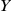 is synthesized when 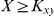 (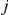 odd), and it is degraded when 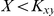 (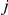 even). The dotted line represents the digital signal 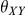. For the plot the following values have been used: 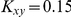, 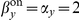, 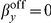.

The solution 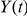 of the ODE (1) is a piecewise function of the form:
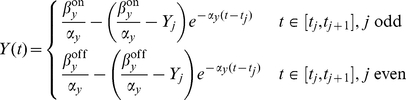
(2)where 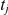 is the 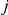-th intersection of the signal with the threshold for 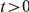, i.e. 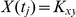, 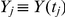, and we assume that 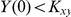. Under the action of a transcription factor 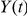 increases exponentially towards 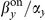 when 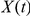 is above the threshold of activation and otherwise decreases exponentially towards the value 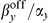 (Figure 2).

Although the specific solution 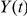 depends on the temporal profile of 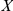 that determines the sequence of on and off times 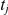, the response of the gene can be characterized by its mean value over a longer time period 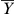. This may also be appropriate for interpreting experimental data from cell populations in which the individual traces of gene expression of single cells are not known and only the population average is measured. When 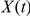 is periodic it can be shown that after a transient time 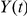 also becomes periodic in time. Moreover in the stationary regime the average value of 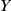 is fully determined by the proportion of time spent by 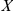 over the activation threshold of gene 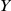 (see Supporting Information S1):
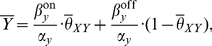
(3)where 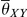 is the time-average of the digital function 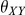. The formula (3) shows that the average value of expression of 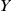 is a weighted average of the equilibrium values that would be attained with no stimulation at all or with constant stimulation. For a signal of given shape, varying the period of oscillation does not change the fraction of time spent over any given threshold, therefore from (3) automatically follows that the average value of 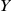 is independent of the period of oscillation of 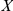. This type of response is described, for example, in Ref. [18] where the expression of genes targeted by Crz1 has the same profile as the frequency of bursts of nuclear Crz1 varying in response to Ca+.

**Figure 3:**
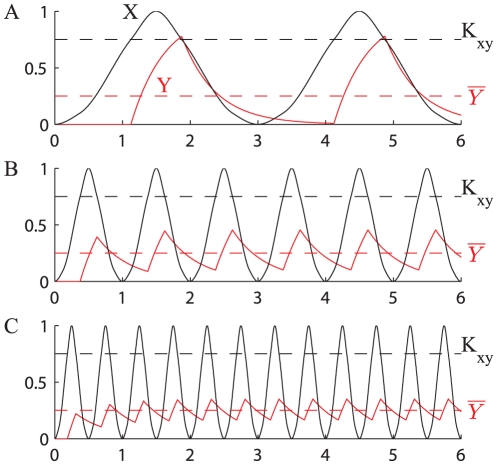
Direct regulation time course dynamics varying the period of 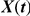. The plots show the response of a gene 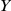 (red curve) and its average value at stationary regime (red dashed) in the case of direct regulation by a symmetric oscillating transcription factor 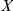 (black curve) having a period of 3 hrs (A) 1 hr (B) 0.5 hr (C). 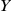 oscillates with varying amplitude depending on the period of oscillation of 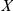. As the frequency of oscillation of 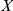 increases, the time 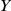 has to adjust decreases, leading to smaller amplitude of its oscillations. The parameters are: 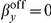, 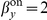, 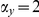, 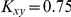 (black dashed, the value has been chosen to activate the production of 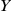 for 25% of time).

**Figure 4:**
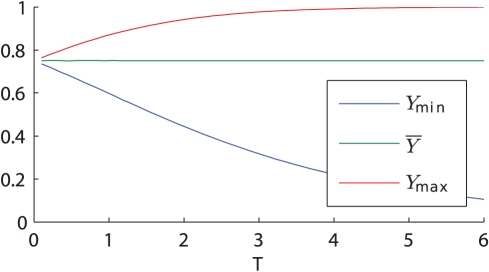
Direct regulation. The minimum (blue) maximum (red) and average (green) values of a transcription factor 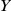 controlled by direct regulation at stationary regime, corresponding to different values of the period of oscillation of 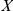. Simulations have been run using 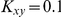, 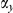 = 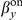 = 1.5, 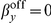.

When the concentration of the oscillatory transcription factor crosses the threshold of activation back and forth only once in each cycle of oscillation (as is typically the case, e.g. NF-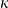
B [27]), it is possible to determine the maximum and minimum values of 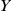 in the stationary regime as:
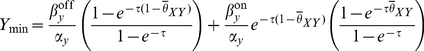
(4)

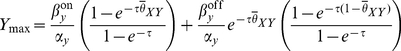
(5)where we defined 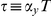 the non-dimensional oscillation period of 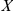 measured relative to the degradation time of 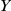. 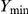 is an increasing function of the period of oscillation and 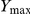 is decreasing, and they both tend to the average value 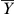 as 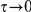. Thus, for a gene that is directly controlled by a single oscillatory transcription factor, although variations in the minimum and maximum level of expression occur (see Figure 3), its average value does not respond to changes in the frequency of oscillations (see Figure 4) or in the shape of the periodic signal as long as the overall percentage time of gene activation remains the same.

### Two-step regulation

The simplest extension of the direct regulation model is the case in which 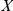 directly regulates 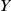 that in turn regulates a third gene 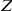. Similarly to the direct regulation case, we assume that 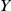 changes the synthesis rate of 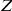 from 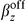 to 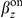 when its concentration is above the threshold 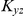, and that 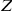 is degraded following a mass-action law with coefficient 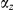.

**Figure 5:**
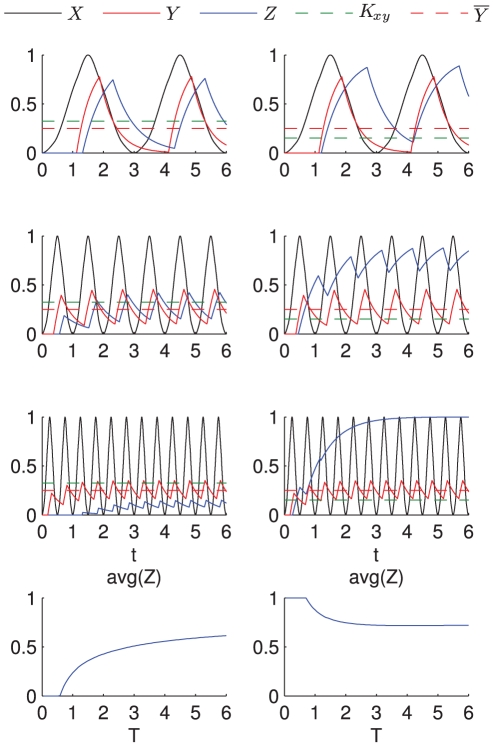
Two-step regulation time course dynamics varying the period of 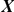. The plots show the expression of a gene 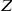 (blue) controlled by the symmetric signal 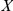 (black) for the two-step model. The left (right) column shows the case in which the value of 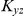 (green dashed line) is above (below) the average value at stationary regime of the transcription factor 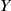 (red curve). The first three rows show the time-course dynamics for 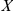, 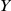 and 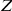 for three different oscillation periods, whereas the last row shows the average value of 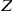 at stationary regime as a function of the period of oscillation of 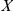. 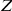 is turned off in the case 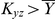 as the period of oscillation decreases (left column) while it is increasingly expressed in the case 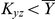 (right column). The parameters used in the simulations are: 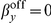, 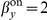, 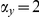, 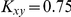 (this value has been chosen so that 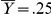) and 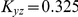 for the left column and 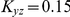 for the right column.

In the case of direct regulation we have shown that the period of oscillation of 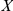 influences the minimum and maximum concentration of 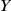 (4–5). Therefore the proportion of time spent by 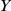 above or below the threshold 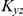 in each cycle of oscillation, i.e. the proportion of time when the expression of 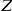 is activated, varies as well in response to changes in the period of oscillation of 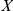 (Figure 3). Since changing the period of 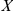 has opposite effects on the minimum and maximum expression levels of 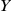 (Figure 4) we can have two types of period dependent responses in the two-step regulation system, depending on the value of the threshold 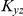. When the threshold is higher than the average concentration of 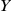, 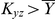, the expression of 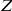 is sensitive to the maximum value of 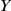, that decreases when the period is shortened and eventually 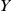 can no longer activate 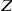. Thus, in this case the average concentration of 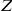 decreases as the period of the input signal 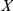 is reduced, and its expression is switched off completely below a certain oscillation period. Conversely if 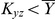, the expression of 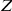 is controlled by the minimum value of 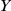, and as the oscillation period of 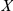 is decreased 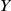 spends more and more time over the value 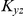 till eventually 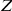 is fully expressed (Figure 5). Thus, in the two-step gene regulatory system, changing the frequency of the input signal can have opposite effects on the expression of genes with different activation thresholds.

**Figure 6:**
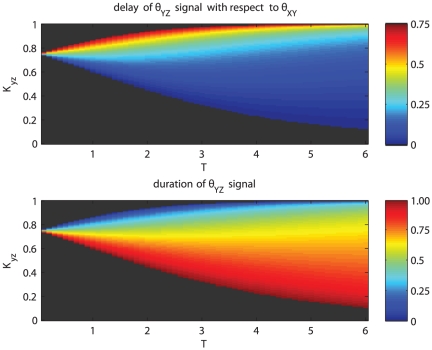
Y expression versus normalized time. The top plot shows the percentage delay in 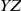 signal with respect to the 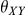 signal, for varying period of oscillation 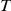 and threshold value 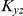, the bottom plot shows the percentage duration of the 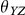 signal. For values of the threshold 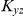 lower than the average value of 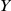, decreasing the period of oscillation causes a faster response (lower delay) and a higher duration of 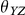 (that eventually is active all the time when the period is small enough). For values of the threshold 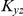 higher than the average value of 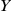, 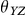 is usually delayed and of short duration, decreasing the period of oscillation causes the maximum value of 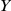 to fall below 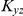 leading to no activation of 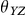. For the plots the parameters 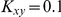, 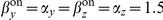, 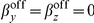 have been used.

The delay occurring between the activation of gene 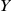 by the transcription factor 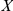 and the activation of 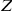 by 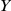 can be evaluated from the time-dependent concentration profile of 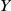 in the increasing branch of (2), i.e. when 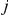 is odd, by finding the value 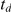 such that 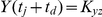. After non-dimensionalization, using again the characteristic lifetime of 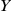 as time unit, we obtain
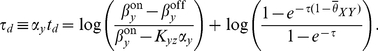
(6)The time between the inactivation of the 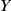 and 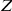 genes can also be obtained with similar calculations, and combining these expressions together the fraction of time spent by the transcription factor 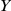 over the threshold 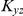 can be evaluated as:
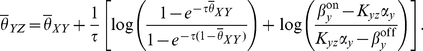
(7)This expression is valid provided that 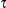 is such that 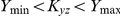. Otherwise, either 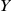 activates the gene 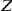 all the time so that 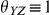 and 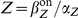, or 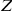 is never activated, i.e. 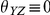 and 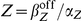. From (7) we can see that depending on the sign of the two logarithmic terms, that can be either positive or negative, the activation time of the target gene 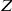 can be either longer or shorter than the time of activation of the intermediate transcription factor 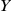. Figure 6 shows how the delay and the duration of the activation change depending on 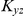 and the period 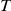 for the same set of parameters as the example given in Figure 5. For a fixed period increasing the activation threshold 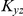 reduces the activation time of 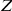. When the period is varied for a given threshold, the activation time, and consequently the average concentration of 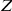 changes monotonously with 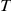 either increasing, when 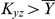, or decreasing otherwise.

To conclude, in the two-step regulation the average expression of the gene 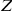 is dependent on the period of the oscillatory input signal 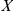. Oscillatory signals with different shapes activating 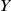 for the same fraction of time, produce a 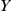 signal that oscillates between the same minimum and maximum values, therefore have no influence on the activation time of 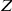. Thus, the average concentration of 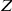 is determined by 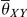, the fraction of time when the input signal is above the activation threshold of the directly regulated gene 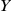 and the period of the input signal.

### Feed-forward loops

**Figure 7:**
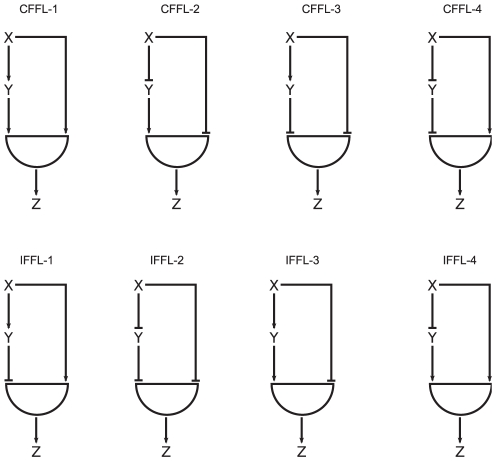
Different types of feed forward loops.

In a FFL the transcription factor 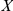 regulates the target gene 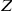 both directly and indirectly through an intermediate transcription factor 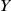 that in turn regulates the transcription of 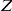. Each of the interactions between the transcription factors 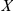, 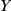 and 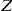 can be either activating or inhibitory, so there are eight different possible combinations as shown in Figure 7. These can be split into two categories: in Coherent Feed Forward Loops (CFFLs) 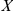 regulates 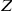 in the same way both directly and indirectly (that is 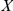 activates or inhibits 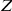 to some extent, through both branches) and Incoherent Feed Forward Loops (IFFLs) in which 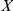 activates 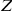 through one branch and inhibits it through the other. The transcription of gene 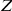 controlled by the FFL is activated by a logic gate, that encapsulates various processes such as DNA binding, RNA polymerase recruitment and so on [2], combining the concentrations of the transcription factors 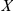 and 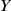 into the expression of 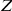. For example, an AND gate in the case of CFFL-1 activates the expression of 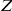 when the concentrations of both 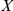 and 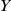 are higher then their separate activation thresholds for 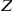, 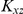 and 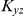. In the case of an IFFL-1 an AND gate allows the transcription of 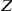 only when the concentration of the activating factor 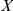 is above its direct regulatory threshold 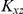, and the inhibitor concentration 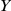 is below the threshold 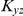. The OR gate activates the expression of 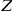 when at least one of the two branches are activated. Several properties of such FFLs have been characterized and tested [1], [31] mostly in the case of step-function type stimulus, here we investigate the properties of FFL motifs when the input signal is an oscillatory transcription factor using the methodology first introduced by Alon in [2, Chap. 4]. In the following we discuss two representative types of FFLs, the CFFL-1 and the IFFL-1, both with AND gates. The other types produce qualitatively similar behavior, just the conditions corresponding to different regimes are interchanged according to the type of interactions between the components. The case of OR gate is also similar and is discussed in Supporting Information S1 using CFFL-1 and IFFL-1 as prototypes for our analysis.

### CFFL-1

**Figure 8:**
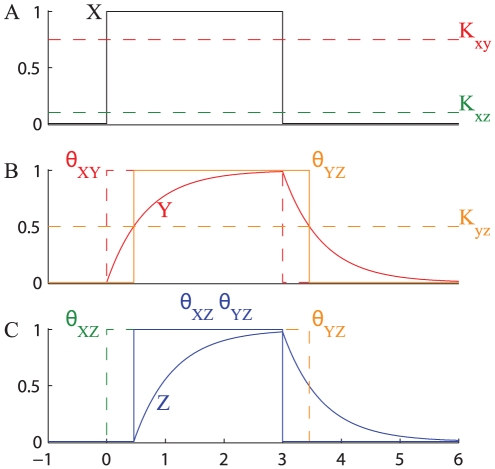
Response of CFFL-1 with AND gate to a step-like stimulation. The figure shows the typical response of a CFFL-1 with AND gate to a transient transcription factor 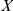. A step-like transcription factor 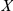 activates simultaneously both branches of the FFL (**A**). Under the stimulus of 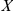 (signal 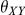), 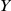 starts accumulating (**B**), but the transcription of 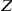 is delayed until 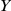 reaches the value 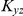, and the signal 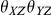 turns on (C). 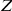 accumulates, but its synthesis is immediately turned off when 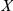 is removed (C). The following parameters have been used: 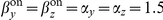, 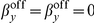, 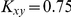, 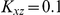, 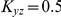.

The activation and inactivation of a CFFL-1 with a step-function on-off stimulus is shown in Figure 8. At time 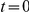 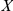 crosses both thresholds 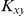 and 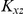 and activates the expression of 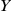 that starts accumulating, and when its concentration reaches 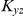 the condition 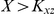 AND 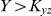 is satisfied and the expression of 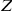 is activated. When the input signal 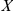 is switched off (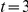), 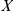 falls below the threshold 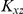 and although the concentration of 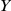 is still higher than 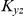 the transcription of 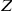 stops instantly. Thus, this type of FFL produces a sign-sensitive delay, i.e. the response is delayed at the on signal but there is no delay when the stimulus is switched off.

**Figure 9:**
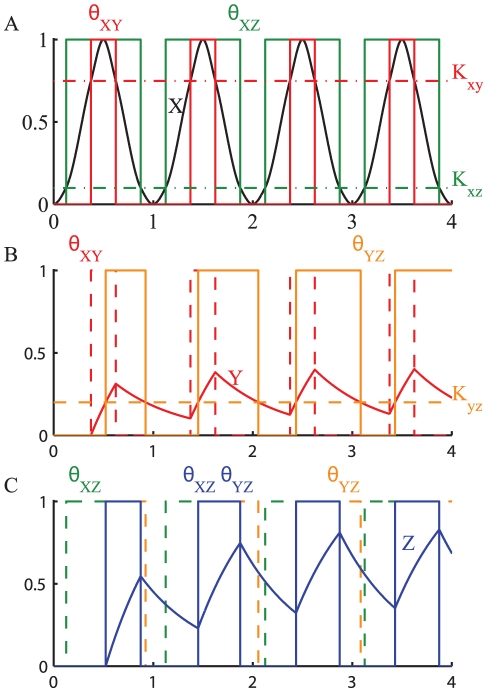
Oscillating transcription factor controlling the expression of gene 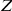 by a CFFL-1 with AND gate. The plot shows the response of a gene expressed under the stimulus of the symmetric signal 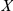 oscillating with period 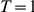. The thresholds 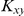 and 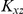 split the signal 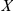 into the two digital signals 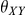 and 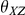 (**A**). 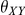 controls the expression of 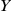 that when over the value 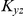 generates the digital signal 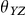 (**B**). The digital signals 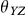 and 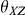 are combined by the logic gate to finally control the expression of the gene 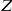, in the case of an AND gate the logic gate only allows the expression of 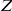 when both 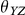 AND 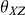 are active (**C**). The parameters used in the simulation are: 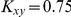, 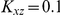, 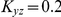, 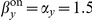, 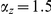, 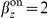, 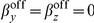.

In the case of an oscillating transcription factor, one branch acts in the same way as explained in the two-step regulation model, but the expression of 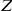 is also influenced by 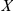 directly on the other branch. Figure 9 shows how the activation of the two branches, combined together by the logic gate, regulates the expression of 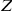. The relative values of 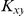 and 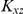 determine which of the two branches is activated first in each cycle of oscillation and the times that 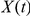 spends above the thresholds 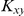 and 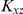, respectively. In the case shown in Figure 9 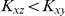, so that 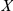 first crosses the threshold 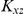 activating the signal 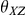 and after some time it goes above 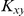 activating also the signal 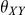. As 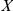 decreases the two branches are deactivated in opposite order, hence 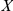 activates the direct branch for a longer time than the indirect one.

**Figure 10:**
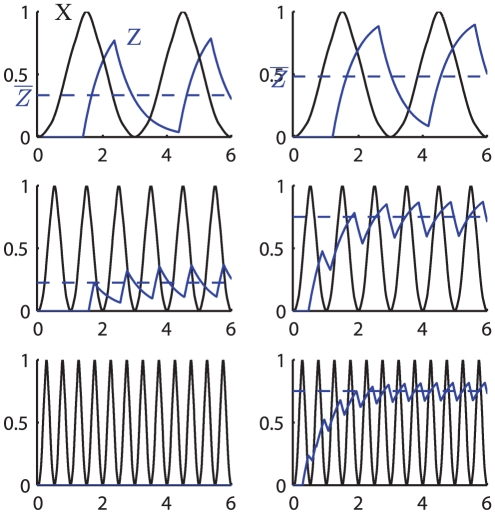
Time course simulation of CFFL-1. The plots show the dynamics of 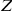 controlled by a CFFL-1 with AND gate stimulated by the symmetric signal 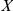 with varying period when 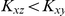. The left column illustrate the case 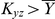, the right column the case 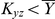. As explained in the main text in the case 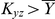 the duration of the 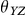 signal diminishes causing the gene 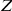 to be inhibited, while when 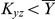 the duration of 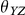 increases causing an increase in the average value of 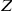. The response is different from the two-step regulation response because the presence of the direct branch limits the maximum duration of the expression of 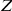. The parameters 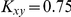 (corresponding to 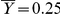), 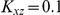, 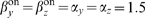, 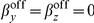 have been used for all the plots. For the left column 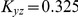; for the right column 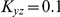.

From the analysis of the two-step regulation model we know that the duration of activation of 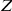 by 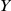 varies with the period of oscillation of 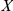. In the case of a CFFL-1 this means that the signal 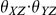, and consequently the average value of 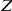, also depend on the period of oscillation of 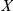. The value 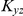 determines whether the duration of activation of 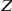 by 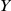 increases (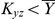) or decreases (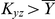) with the period of oscillation. As in the two-step regulation case if 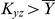 the average value of 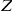 is eventually switched off as the frequency of oscillation increases (Figure 10, left column). If 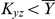 the average value of 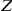 increases as the frequency of oscillation increases. However in contrast with the two-step regulation 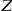 is never fully expressed. Even when the minimum of 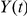 is above the threshold of activation of 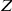, the expression of 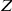 is still limited by the activation of the direct branch. The value of 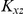, that determines the fraction of time when 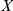 directly activates 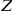, sets a limit to the maximum average concentration of 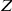 (Figure 10, right column).

The period of oscillation and the temporal profile of 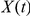 also influence the delay between the signals 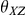 and 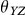. This suggests that the shape of the signal 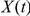 can play a role in controlling the expression of 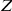. If the oscillations of 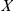 are skewed to the left, i.e. steep increase followed by slower decay, then 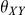 and 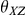 are activated almost simultaneously, but one of them is deactivated well before the other, conversely if 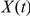 is skewed to the right, one of the two signals is activated before the other and they are deactivated almost at the same time. The delay of 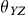 with respect to 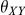 due to the time required for the accumulation of 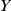, can results in out of phase activation of 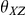 and 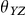, reducing the duration of activation of the gene 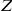. The effects of the shape of the signals is discussed further below.

**Figure 11:**
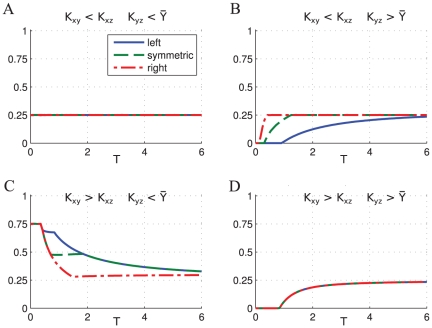
CFFL-1 AND gate, average response of the gene 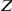 at stationary regime for various configurations of the parameters. For all the plots the values 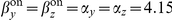 have been used. The thresholds of activation for the various cases are: (**A**) 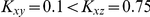, 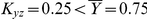; (**B**) 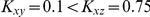, 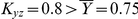; (**C**) 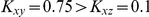, 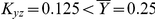; (**D**) 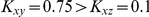, 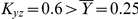.

Varying the period of the input signal we have identified four different classes of responses, depending on the relative values of the thresholds 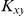, 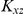, and on whether the indirect regulation of 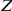 is controlled by the low or high values of the intermediate transcription factor 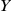, i.e. 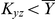 or 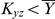. Figure 11 shows the average concentration of 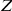 at stationary regime obtained stimulating the CFFL-1 (AND gate) with oscillatory signals of different shapes and varying the oscillation period:

**A)** 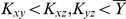. The average level of 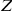 is not affected by the period of oscillation of 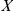 as in the case of direct regulation by a single transcription factor. Since 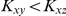, the signal 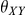 contains 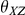 and the accumulation of 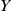 starts before 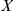 directly activates 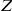. Since 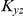 is low, 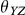 is activated before 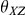, and it is deactivated after 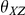 is switched off. The result is that 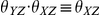 so that 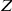 is expressed as if only directly regulated by 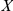, and is independent of the indirect branch. Therefore the average concentration of 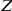 does not change with the frequency, and the shape of the input signal 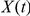 has no effect on the final outcome.

**B)** 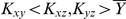. The expression of 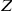 is switched off at high frequency oscillations, then increases with the period and saturates at a value corresponding to the direct activation of 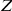 by 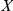. Similarly to the two-step regulation the switch is controlled by the maximum value of 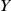 that for high frequency oscillations falls below the threshold 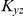, that switches off the expression of 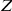. The delay between 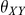 and 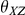 also influences the response and shows a gradual switch between activation and inactivation when 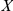 activates 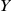 and 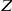 simultaneously, and a sharp transition when the activation of 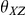 is delayed with respect to 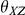 with a delay.

**C)** 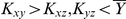. In this case 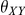 is activated after 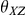. However since 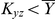 decreasing the period of oscillation causes an increase in the duration of 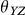, so that 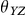 and 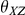 overlap for longer time and the average expression of 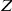 increases. In this case as well, the shape of the input signal influences the response since as the period decreases the signal 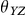 lasts longer causing the duration of the overlap to vary smoothly or abruptly depending on the delay between 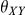 and 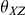.

**D)** 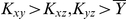. The expression of 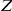 is controlled by the indirect branch through 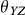, therefore this case is equivalent to the two-step regulation case presented earlier. Since 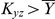 the duration of 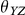 decreases with the decreasing of the period of oscillation until eventually 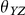 is no longer active. The shape of the signals does not affect the response because the duration of 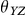 is short compared to 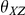 and therefore the variations in the delay do not cause any significant change in the average value of expression.

### IFFL-1

**Figure 12:**
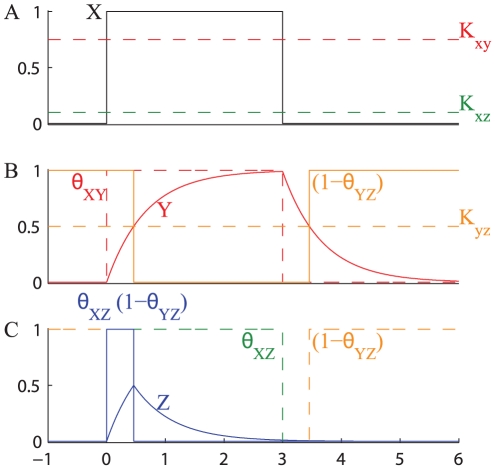
Response of IFFL-1 with AND gate to step-like stimulation. The plots show that under a step-like stimulus an IFFL-1 generates a pulse-like response. When 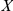 activates the FFL (**A**) the logic gate is activated by 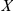 but is not inhibited by 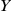 starting the expression of 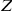. Under the stimulation of 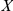, 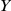 starts to build up, but it only inhibits the expression of 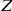 after the delay required to reach the value 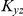 (**B**). As a result 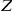 starts decreasing when 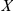 is still present. For the simulation the following parameters have been used: 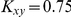, 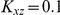, 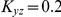, 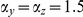, 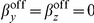, 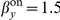, 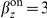.

As a representative of the IFFLs we illustrate the behavior of the IFFL-1 with an AND gate. In the IFFL-1 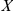 directly promotes the expression of the gene 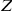 and inhibits it indirectly by activating the expression of the repressor 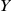. The transcription of 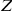 is activated when 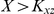 AND 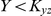, i.e. following to the digital signal 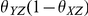. In Figure 12 the activation and inactivation of the IFFL-1 by a constant step-like stimulus is shown. At the time 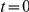 the transcription factor crosses the thresholds 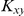 and 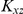 and since 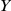 is initially not present, 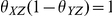, the transcription of 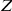 is activated. Meanwhile, 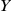 starts accumulating and after a transient time reaches the threshold of inhibition 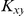 that turns off the expression of 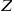. Thus, in the case of a step-like sustained stimulus the IFFL-1 is a *pulse generator* promoting the expression of the gene 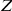 only for a limited time.

**Figure 13:**
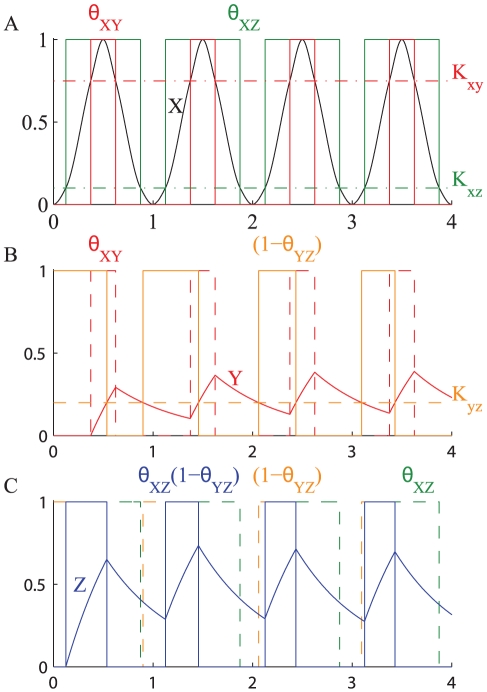
Oscillating transcription factor controlling the expression of gene 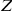 by means of an IFFL-1 with AND gate. The plot shows the response of a gene expressed under the stimulus of an oscillating transcription factor 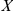. The thresholds 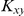 and 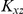 split the signal 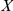 into the two digital signals 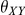 and 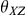 (**A**). 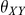 controls the expression of 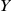 (**B**). The digital signals 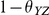 and 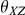 are combined by the logic gate to finally control the expression of the gene 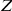 (**C**). For the simulation the following parameters have been used: 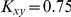, 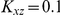, 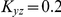, 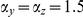, 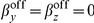, 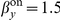, 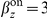.

**Figure 14:**
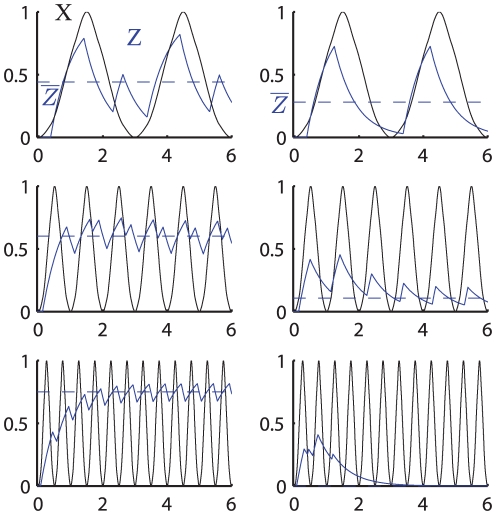
Time course simulation of IFFL-1. The plots show the dynamics of 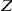 stimulated by an oscillating transcription factor in the two different cases when 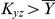 (left column) and 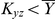 (right column), stimulated with oscillating transcription factors of varying period. As explained in the main text, in the case 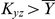 the duration of the 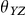 signal diminishes as the frequency of oscillation increases; as a consequence the average value of the signal 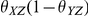 increases and so does the average value of 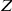. When 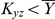, the duration of the 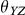 signal increases with the frequency of oscillation and so the average value of 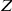 decreases. The values 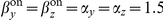, 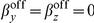, 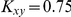, 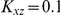 have been used for all the plots. For the left column 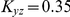, for the right column 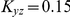.

In the presence of an oscillating factor 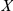, however, the IFFL-1 acts as an oscillation detector, continuously activating and deactivating the expression of 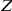, so that the average amount of 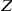 can be high in the presence of sustained oscillations. This is illustrated in Figure 13, using the same parameters as in Figure 12. The oscillating transcription factor 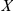 periodically crosses the thresholds 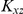 and 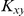 turning on and off the two branches with a certain delay relative to each other. Under the direct regulation of 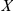, the repressor 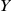 is expressed and degraded crossing back and forth the threshold 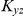, generating the oscillatory signal 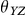. This combined with the direct activation of 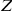 leads to the periodic expression of 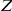 and is not turned off completely after a transient time. Once again while the average amount of 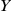 is independent of the period of oscillation of 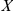, its maximum and minimum values are not, and as a consequence, it affects the signal 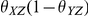 that controls the expression and the average concentration of 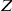. As explained before, depending on the value of 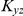, the time spent above the threshold 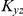 by the concentration of 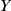 can either increase or decrease with the period of oscillation. In the specific case of the IFFL-1 with an AND gate if 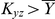 as the period of oscillation of 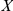 decreases, the time when the repressor 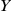 is active decreases as well. As a consequence, the average concentration of 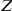 increases when the period of oscillations is increased (Figure 14). Similarly to the CFFL-1 the values of the thresholds determine the relative delays between the activation of the different branches, so that IFFLs can be activated differently by transcription factors 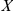 with different temporal profiles.

**Figure 15:**
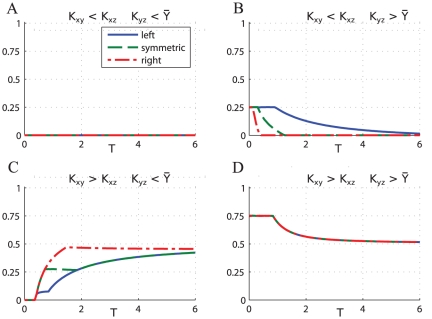
IFFL-1 AND gate, average response of the gene 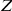 at stationary regime. For all the plots the values 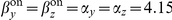 have been used. The thresholds of activation for the various cases are: (**A**) 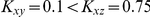, 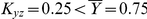; (**B**) 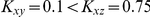, 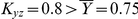; (**C**) 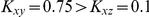, 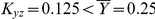; (**D**) 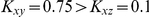, 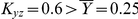.

The response to changing the period of the oscillation of 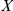 can be classified again into four different regimes as shown in Figure 15:

**A)** 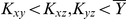 In this case the signal 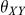 contains 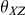. Since the threshold of activation is low, the repressor 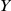 is already active when 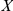 activates 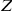. Since the activity of the repressor completely overlaps with the direct activation, the gene 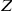 is not expressed regardless of the oscillation period.

**B)** 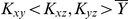 In this case the average expression of 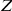 decreases as the period increases. Since 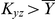 the duration of the activity of the repressor decreases as the frequency of the oscillation increases, until eventually the maximum concentration of 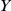 falls below the threshold 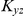 and the repressor 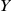 is completely switched off. The relative delay between the direct activation and the activation of the repressor in this case influences the final outcome since it determines whether 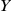 has sufficient time to accumulate to reach the threshold of inhibition. This is shown by the distinct response functions obtained for the signals with different temporal profiles of the oscillatory input signals.

**C)** 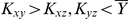 In this configuration of the parameters the signal 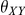 is contained in 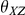. Since the threshold 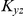 is low, 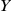 inhibits the transcription of 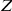 almost immediately after its expression is activated by 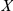, limiting the transcription activated directly by 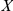. As the frequency of oscillation increases 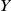 spends more and more time over the threshold inhibiting 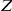 for longer times, until eventually completely switches off the expression of 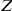. The relative delay between 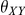 and 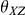 determines the sharpness of the transition.

**D)** 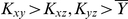 Since the threshold of inhibition 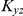 is high, activation of the repressor 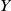 needs some time. When the period of 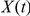 is large enough, 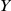 reaches the threshold inhibiting 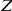, but as the period of oscillation decreases 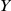 does not have time to accumulate and its inhibitory effect ceases. Also, since the threshold of activation is high, the variations in the delay and length of 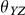 are small compared to the duration of 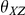 and the shape of the signal does not influence the average synthesis rate of the gene 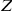.

## Discussion

Oscillations are a widespread phenomenon arising in many biological systems [32]. Gene expression however has been mostly studied as a static phenomenon mainly focussing on the total amount of transcription factor activated by various types of stimuli, usually observed after a relatively long treatment. This approach allows to infer information about the processes ongoing in the cell at population level, but does not provide insight into the dynamics of the components involved and about their influence on the final outcome of gene expression. Nevertheless high-throughput experiments have started to unravel the complexities of temporal dynamics and have shown the importance of understanding the information encoded in the temporal dynamics of cellular processes.

Previous studies have investigated both theoretically and experimentally the properties of regulatory networks in relation to their topology. Alon and coworkers have demonstrated various properties of simple regulatory motifs like negative auto-regulation [4] and feed forward loops [2], [6], [9], [12]–[15]. In [33] the authors have shown by means of numerical simulations and linear analysis that the presence of common three and four node motifs could be beneficial for the robustness of biological networks to small perturbations and noise. The dynamic response to bursts of activation for common motifs like IFFL-1, diamond-motif and the interlocked negative loop was studied in [8] by characterizing the optimal duration of inter-pulse intervals that maximizes the time-averaged response.

Under adequate stimulation oscillations in gene expression may involve a large number of transcription factors, propagating across different pathways and occur at different cellular levels [28]–[30]. In principle, such background oscillations allow for refined context-dependent activation of pathways in response to different specific stimuli. For example, recent work on the NF-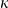
B pathway has shown that the oscillations caused by the negative feedback loop through IkB family of proteins and A20, are tightly regulated and suggest a frequency as well as amplitude dependence of the transcription of targeted genes, although the mechanisms of differential response by means of oscillations has not been clarified yet. 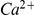 dependent bursts of nuclear Crz1 in yeast and bacteria has shown that oscillation in and out the nucleus can be advantageous for maintaining the relative amount of certain proteins constant in the cell [18].

In this work we studied the possibility of frequency dependent responses in simple gene regulatory schemes, that could be used in decoding information from time-dependent oscillatory signals, and to generate differential regulation of multiple genes controlled by the temporal dynamics of the same transcription factor.

In the case of direct regulation the key factor regulating the gene expression is the fraction of time when the transcription factor concentration is above the activation threshold of a certain gene. As a consequence, modifying the frequency of oscillation cannot modulate the expression of a gene. Varying the amplitude of oscillation though, may cause changes in the duration of the activity of transcription factors and could regulate the average level. Such a mechanism might be ideal to regulate those genes whose average level of expression in cells and tissues should not change when the cellular environment is perturbed by a stimulus that gives rise to oscillations.

For the two-step regulation the frequency of oscillation is capable of switching on or off the expression of the target genes. Increasing the frequency of oscillation of the regulating transcription factor causes the intermediate component to oscillate closer to its average value 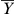. As a consequence, depending on the threshold of activation of the target genes they could be up or down-regulated in a frequency dependent manner. However, since the input signal activates gene expression by crossing over a single threshold, this mechanism cannot distinguish between different temporal profiles of the transcription factor. This is possible for feed forward loops when the input signal activates two different genes with different activation thresholds.

Thus increasing the complexity of the gene regulatory network provides the cell with more refined mechanisms for decoding information from the temporal dynamics, that is not possible in the case of steady-state responses with no temporal dynamics. We have identified distinct types of response behaviors depending on the parameters, for example: on/off switching of the gene expression in a frequency dependent manner, maintenance of a constant average expression, frequency dependent switching of the expression level between two distinct regimes. Moreover we have shown that, as 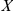 activates the two branches of a FFL at different times depending on the shape of the signal, the temporal profile of 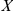 can affect the final average expression of the targeted gene. For our simulations we have used signals that vary between the same maxima and minima and have approximately the same average value, but yet the outcome on gene expression is different. Such a behavior could for example explain why in certain experiments involving cell population measurements, even if the amount of the considered transcription factor is the same in different samples the genetic response can be completely different.

Gene expression mediated by two-step regulation and FFLs could be advantageous in driving cell fate in those situations for which the transcription factor can regulate opposite cellular processes. NF-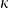
B and p53 for example are known to regulate both apoptosis and cell proliferation. We have shown that different genes may respond differently to the same oscillatory signal depending on the parameters and the topology of the interaction networks. Thus, regulation of such different cell fates may be possible by encoding certain environmental information in the frequency of oscillations of NF-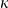
B so that certain genes favoring one process or the other become activated.

Future extensions of this work could consider how combining together several of these regulatory mechanisms affects the ability to decode information from the temporal dynamics of transcription factors in transcriptional networks with more complex topology. Another interesting possibility would be to consider gene regulatory motifs controlled by oscillatory input signals that depend on multiple stimuli, to explore how multiple information can be transmitted and recovered from the temporal dynamics of a single transcription factor. The inputs influencing the dynamics of an oscillatory transcription factor typically would modify not just the frequency but also other characteristics of the signals, e.g. average expression rate, amplitude of the oscillations etc. Therefore the frequency dependent responses that we described may be combined with or dominated by other changes occurring simultaneously.

While in our models we focussed on the time-averaged response behavior of a stationary oscillating system, in many cases transient signaling and the timing of the gene expression is also important. Relevant information may also be encoded in the temporal profile of transient stimuli, that could lead to selective transient expression of different genes. Simple gene regulatory networks can also play a role in decoding such information as it was shown for example in the context of genes involved in cell cycle regulation [11].

Frequency dependent expression of genes regulated by NF-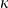
B has been observed experimentally in [34]. In this work oscillations of NF-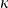
B activity were triggered by stimulating the cells with pulses of the inflammatory stimulus, TNF-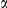, promoting waves of translocation of NF-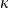
B into the nucleus resulting in differential gene expression, dependent on the period of the external stimulus. NF-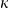
B regulates hundreds of genes whose expression is likely to be interconnected, and therefore this pathway could be a good candidate as a model system for validating our theoretical findings. This could be done for example by identifying groups of genes with qualitatively similar activation patterns in response to changes in the oscillation period, e.g triggered by different concentrations of TNF-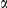. Then the next step would be to find correlations between the different types of frequency-dependent responses with the characteristic gene interaction patterns. Mutant cells in which different forms of I
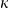
B have been suppressed leading to irregular period of oscillations could also be used to test the effects of oscillation period on a the final outcome of gene expression. Another potential candidate for such experimental work is the oscillatory transcription factor p53 that regulates hundreds of genes whose period of oscillation has been shown to be dependent on the cell type and varies in response to different stimuli [20].

## Supporting Information

Supporting Information S1 This supporting material contains the mathematical derivation of all the formulas presented in the main text. The numerical results obtained simulating Network Motifs with an OR gate stimulated by oscillatory transcription factors are shown and discussed extensively using the same framework introduced in the main text.
(PDF)

## References

[1] Alon U (2007). Network motifs: theory and experimental approaches.. Nature Reviews Genetics.

[2] Alon U (2007). An introduction to systems biology.

[3] Tyson J, Novák B (2010). Functional motifs in biochemical reaction networks.. Annual Review of Physical Chemistry.

[4] Rosenfeld N, Elowitz M, Alon U (2002). Negative autoregulation speeds the response times of transcription networks.. Journal of Molecular Biology.

[5] Isaacs F, Hasty J, Cantor C, Collins J (2003). Prediction and measurement of an autoregulatory genetic module.. Proceedings of the National Academy of Sciences of the United States of America.

[6] Kalir S, Mangan S, Alon U (2005). A coherent feed-forward loop with a sum input function prolongs agella expression in escherichia coli.. Molecular Systems Biology.

[7] Maeda Y, Sano M (2006). Regulatory dynamics of synthetic gene networks with positive feedback.. Journal of Molecular Biology.

[8] Cournac A, Sepulchre J (2009). Simple molecular networks that respond optimally to time-periodic stimulation.. BMC Systems Biology.

[9] Mangan S, Zaslaver A, Alon U (2003). The coherent feedforward loop serves as a sign-sensitive delay element in transcription networks.. Journal of Molecular Biology.

[10] Basu S, Mehreja R, Thiberge S, Chen M, Weiss R (2004). Spatiotemporal control of gene expression with pulse-generating networks.. Proceedings of the National Academy of Sciences of the United States of America.

[11] Csikász-Nagy A, Kapuy O, Tóth A, Pál C, Jensen L (2009). Cell cycle regulation by feed- forward loops coupling transcription and phosphorylation.. Molecular Systems Biology.

[12] Mangan S, Itzkovitz S, Zaslaver A, Alon U (2006). The incoherent feed-forward loop accelerates the response-time of the gal system of escherichia coli.. Journal of Molecular Biology.

[13] Goentoro L, Shoval O, Kirschner M, Alon U (2009). The incoherent feedforward loop can provide fold-change detection in gene regulation.. Molecular Cell.

[14] Shoval O, Goentoro L, Hart Y, Mayo A, Sontag E (2010). Fold-change detection and scalar symmetry of sensory input fields.. Proceedings of the National Academy of Sciences.

[15] Kaplan S, Bren A, Dekel E, Alon U (2008). The incoherent feed-forward loop can generate non- monotonic input functions for genes.. Molecular Systems Biology.

[16] Aulehla A, Pourquié O (2008). Oscillating signaling pathways during embryonic development.. Cur- rent Opinion in Cell Biology.

[17] Dolmetsch R, Lewis R, Goodnow C, Healy J (1994). Differential activation of transcription factors induced by Ca2+ response amplitude and duration.. Annu Rev Genet.

[18] Cai L, Dalal C, Elowitz M (2008). Frequency-modulated nuclear localization bursts coordinate gene regulation.. Nature.

[19] Locke J, Elowitz M (2009). Using movies to analyse gene circuit dynamics in single cells.. Nature Reviews Microbiology.

[20] Batchelor E, Loewer A, Lahav G (2009). The ups and downs of p53: understanding protein dynamics in single cells.. Nature Reviews Cancer.

[21] Geva-Zatorsky N, Rosenfeld N, Itzkovitz S, Milo R, Sigal A (2006). Oscillations and variability in the p53 system.. Molecular Systems Biology.

[22] Hirata H, Yoshiura S, Ohtsuka T, Bessho Y, Harada T (2002). Oscillatory expression of the bhlh factor hes1 regulated by a negative feedback loop.. Science.

[23] Nelson D, Ihekwaba A, Elliott M, Johnson J, Gibney C (2004). Oscillations in NF-*κ*B signaling control the dynamics of gene expression.. Science.

[24] Cheong R, Hoffmann A, Levchenko A (2008). Understanding NF-*κ*B signaling via mathematical modeling.. Molecular Systems Biology.

[25] Paszek P, Jackson D, White M (2010). Oscillatory control of signalling molecules.

[26] Tay S, Hughey J, Lee T, Lipniacki T, Quake S (2010). Single-cell nf-*κ* b dynamics reveal digital activation and analogue information processing.. Nature.

[27] Sung M, Salvatore L, De Lorenzi R, Indrawan A, Pasparakis M (2009). Sustained oscillations of nf-*κ*b produce distinct genome scanning and gene expression profiles.. PLoS One.

[28] Sun L, Yang G, Zaidi M, Iqbal J (2008). TNF-induced gene expression oscillates in time.. Biochemical and Biophysical Research Communications.

[29] Sun L, Yang G, Zaidi M, Iqbal J (2008). TNF-induced oscillations in combinatorial transcription factor binding.. Biochemical and Biophysical Research Communications.

[30] Iqbal J, Zaidi M (2008). TNF-induced MAP kinase activation oscillates in time.. Biochemical and Biophysical Research Communications.

[31] Mangan S, Alon U (2003). Structure and function of the feed-forward loop network motif.. Proceedings of the National Academy of Sciences of the United States of America.

[32] Kruse K, Julicher F (2005). Oscillations in cell biology.. Current Opinion in Cell Biology.

[33] Prill R, Iglesias P, Levchenko A (2005). Dynamic properties of network motifs contribute to biolog- ical network organization.. PLoS Biology.

[34] Ashall L, Horton C, Nelson D, Paszek P, Harper C (2009). Pulsatile stimulation determines timing and specificity of nf-*κ*b-dependent transcription.. Science.

