# Correction: Differential Gene Expression Regulated by Oscillatory Transcription Factors

**DOI:** 10.1371/annotation/99045f74-9bb1-4bde-bdc0-876ee52b5025

**Published:** 2012-10-03

**Authors:** Luca Cerone, Zoltán Neufeld

Figure 15 is incorrect in the PDF. It is correct in the online version of the article.

