# Supplementary material for: Differential Gene Expression Regulated by Oscillatory Transcription Factors
Source: PLoS One. 2012 Jan 24;7(1):e30283. doi: 10.1371/journal.pone.0030283 (PMC3265475; doi:10.1371/journal.pone.0030283)
Supplement: Supporting Information S1 — This supporting material contains the mathematical derivation of all the formulas presented in the main text. The numerical results obtained simulating Network Motifs with an OR gate stimulated by oscillatory transcription factors are shown and discussed extensively using the same framework introduced in the main text. (PDF) [file pone.0030283.s001.pdf]

Supporting Information S1

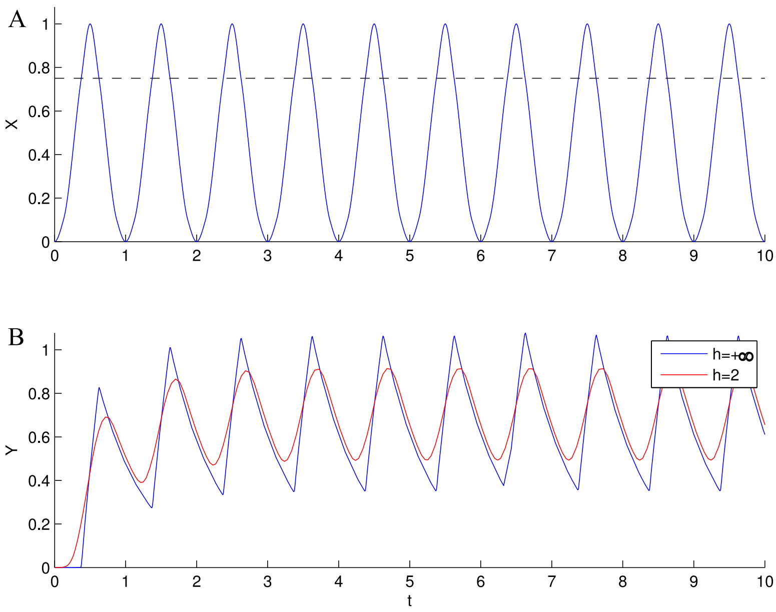

Figure S1. Step-like activation threshold VS Hill function. The plot B shows the numerical simulations for a gene directly regulated by an oscillating transcription factor obtained solving the ODE (1) in the main text, that is using a Hill function for the synthesis in the limit case when $h \to \infty$ (blue) and one obtained using a Hill coefficient h=2 (red), when stimulated with the symmetric signal X described in the main text. Except for the values of h the other parameters are the same in both cases, i.e. $\beta_y^{\text{on}} = 4$, $\alpha_y = 1.5$ and $K_{xy} = .75$. Although the dynamics is slightly different, the qualitative behavior is very similar and the limit case ($h \to \infty$) is a good approximation to study the behavior of transcriptional regulation.

Mathematical proof that Y attains a stationary regime

We assume that $X(t)$ is a continuous function of period T, i.e. that the function X repeats itself at regular intervals of time T. To prove that the function $Y(t)$ attains a stationary regime, we consider the sequence of values of $Y_0 = \beta_{off}/\alpha_y, Y_1 = Y(T), \ldots Y_n = Y(nT), \ldots$ that is the sequence of values of Y at the beginning of each cycle of oscillation, having assumed without loss of generality that X goes above the threshold for the first time at $t = 0$) and show that it converges, so that $Y(t)$ will be periodic itself.

Let's first notice that the function $f(x) = c - (c - x)\exp(-\delta)$ is such that its derivative $f(x)' = 0 < \exp(-\delta) < 1, \forall x, c, \delta > 0$. As $X(t)$ crosses the threshold K_{xy} in each cycle of oscillation it determines k intervals in each of which the function Y is defined by a function f_i having the same form as $f(x)$ and different values of c and δ. The sequence Y_n can than be defined iteratively as $Y_n = F(Y_{n-1}) = f_k(f_{k-1}(\ldots(f_1(Y_{n-1}))))$. By the rule of derivation of a composed function we can then conclude that $0 < F'(X) < 1$, meaning that the sequence can be described by iterating a Lipschitz continuous function with Lipschitz constant $L < 1$. From calculus we know that such a sequence is convergent and as a consequence Y attains a stationary regime.

Mean value of $Y(t)$.

In the previous section we have shown that under the action of a periodic transcription factor X, Y attains a stationary regime. Here we show that the average value of Y is given by the formula (3) presented in the main text.

From the definition of the ODE (1), by integrating both sides of equation with respect to t in the interval $[nT;(n+1)T]$, assuming that n and Y is at stationary regime ($Y_{n+1} = Y_n$), we get

$$Y_{n+1} - Y_n = 0 = \int_{nT}^{(n+1)T} \beta_y^{\text{off}} + (\beta_y^{\text{on}} - \beta_y^{\text{off}})\theta_{XY}(t)dt - \alpha_y \int_{nT}^{(n+1)T} Y(t)dt$$

,

Rearranging the terms and dividing both sides by T then

$$\overline{Y} = \frac{1}{T}\int_{nT}^{(n+1)T} Y(t)dt = \frac{1}{T}\int_{nT}^{(n+1)T} \frac{\beta_y^{\text{off}} + (\beta_y^{\text{on}} - \beta_y^{\text{off}})\theta_{XY}(t)}{\alpha_y} dt =$$
$$= \frac{1}{T}\left(\frac{\beta_y^{\text{on}}}{\alpha_y}\sum_{i=0}^{k}(t_{2i+1} - t_{2i}) + \frac{\beta_y^{\text{off}}}{\alpha_y}\sum_{i=0}^{k}(t_{2(i+1)} - t_{2i+1})\right) =$$
$$\frac{\beta_y^{\text{on}}}{\alpha_y}\overline{\theta}_{XY} + \frac{\beta_y^{\text{off}}}{\alpha_y}(1 - \overline{\theta}_{XY})$$

where $\overline{\theta}_{XY}$ is the fraction of time spent over the threshold K_{xy} by $X(t)$.

Derivation of the expressions for Y_{min} and Y_{max}

In the case when the oscillating concentration of transcription factor X crosses the threshold of activation K_{xy} only two time per cycle, then the period T is split into two intervals, corresponding to the times above and below the threshold, respectively. Assuming that the cycle begins when X goes above the threshold of activation, evaluating the minimum of Y is equivalent to evaluating the value of Y at the beginning of each cycle in the stationary regime, that is we have to evaluate the fixed point of the function $F(x)$ that maps Y at the beginning of the n-th cycle into the one it has at the beginning of the $(n+1)$-th cycle.

The function $F(x)$ in this case is

$$F(x) = \frac{\beta_y^{\text{off}}}{\alpha_y} - \left(\frac{\beta_y^{\text{off}}}{\alpha_y} - \left(\frac{\beta_y^{\text{on}}}{\alpha_y} - \left(\frac{\beta_y^{\text{on}}}{\alpha_y} - x\right)e^{-\tau\overline{\theta}_{XY}}\right)\right)e^{-\alpha_y(1-\overline{\theta}_{XY})}$$

and the fixed point can be evaluated by solving

$$Y_{\text{min}} = F(Y_{\text{min}}) = \frac{\beta_y^{\text{off}}}{\alpha_y} - \left\{\frac{\beta_y^{\text{off}}}{\alpha_y} - \left[\frac{\beta_y^{\text{on}}}{\alpha_y} - \left(\frac{\beta_y^{\text{on}}}{\alpha_y} - Y_{\text{min}}\right)e^{-\tau\overline{\theta}_{XY}}\right]\right\}e^{-\tau(1-\overline{\theta}_{XY})}$$

rearranging the terms we obtain:

$$Y_{\text{min}}\left(1 - e^{-\tau}\right) = \frac{\beta_y^{\text{off}}}{\alpha_y}\left(1 - e^{-\tau(1-\overline{\theta}_{XY})}\right) + \frac{\beta_y^{\text{on}}}{\alpha_y}e^{-\tau(1-\overline{\theta}_{XY})}\left(1 - e^{-\tau\overline{\theta}_{XY}}\right)$$

hence

$$Y_{\text{min}} = \frac{\beta_y^{\text{off}}}{\alpha_y}\left(\frac{1 - e^{-\tau(1-\overline{\theta}_{XY})}}{1 - e^{-\tau}}\right) + \frac{\beta_y^{\text{on}}}{\alpha_y}e^{-\tau(1-\overline{\theta}_{XY})}\left(\frac{1 - e^{-\tau\overline{\theta}_{XY}}}{1 - e^{-\tau}}\right)$$

The value Y_{max} can be evaluated as the value that Y has, when X stops activating the expression of Y, that is

$$Y_{\text{max}} = \frac{\beta_y^{\text{on}}}{\alpha_y} - \left(\frac{\beta_y^{\text{on}}}{\alpha_y} - Y_{\text{min}} \right) e^{-\tau \bar{\theta}_{xy}}$$

By substituting the value of Y_{min} one finds that

$$Y_{\text{max}} = \frac{\beta_y^{\text{on}}}{\alpha_y} \left(\frac{1 - e^{-\tau \bar{\theta}_{XY}}}{1 - e^{-\tau}} \right) + \frac{\beta_y^{\text{off}}}{\alpha_y} e^{-\tau \bar{\theta}_{XY}} \left(\frac{1 - e^{-\tau(1-\bar{\theta}_{XY})}}{1 - e^{-\tau}} \right)$$

Derivation of the relative delay between Y and Z

Here we show how to evaluate the delay between the activation of Y and the activation of Z by Y (equation (6) of the main text).

In the following we assume that the system has attained a stationary regime and, without loss off generality, we consider that X crosses the threshold value K_{xy} at the time $t_j = 0$. As X goes above the threshold, Y starts accumulating according to the formula

$$Y(t) = \frac{\beta_y^{\text{on}}}{\alpha_y} - \left(\frac{\beta_y^{\text{on}}}{\alpha_y} - Y_{\text{min}} \right) e^{-\alpha_y t}$$

and the delay t_d can be evaluated by finding the value t for which $Y(t_d) = K_{yz}$. The non dimensional expression for τ_d then simply follows by considering $\alpha_y t_d$. From $K_{yz} = Y(t_d)$, rearranging the terms one gets:

$$e^{-\alpha_y \cdot t_d} = \frac{K_{yz} - \frac{\beta_y^{\text{on}}}{\alpha_y}}{Y_{\text{min}} - \frac{\beta_y^{\text{on}}}{\alpha_y}}$$

hence

$$\tau_d = \alpha_y t_d = -\log \left(\frac{K_{yz} - \frac{\beta_y^{\text{on}}}{\alpha_y}}{Y_{\text{min}} - \frac{\beta_y^{\text{on}}}{\alpha_y}} \right) = \log \left(\alpha_y Y_{\text{min}} - \beta_y^{\text{on}} \right) - \log \left(\alpha_y K_{yz} - \beta_y^{\text{on}} \right) \tag{1}$$

Using equation (4) we obtain that

$$\alpha_y Y_{\text{min}} - \beta_y^{\text{on}} = (\beta_y^{\text{off}} - \beta_y^{\text{on}})(1 - e^{-\tau(1-\bar{\theta}_{XY})})/(1 - e^{-\tau})$$

so that equation (1) becomes

$$\tau_d = \log \left(\beta_y^{\text{off}} - \beta_y^{\text{on}} \right) - \log \left(\alpha_y K_{yz} - \beta_y^{\text{on}} \right) + \log \left(1 - e^{-\tau(1-\bar{\theta}_{XY})} \right) - \log \left(1 - e^{-\tau} \right) =$$

$$= \log \left(\frac{\beta_y^{\text{on}} - \beta_y^{\text{off}}}{\beta_y^{\text{on}} - \alpha_y K_{yz}} \right) + \log \left(\frac{1 - e^{-\tau(1-\bar{\theta}_{XY})}}{1 - e^{-\tau}} \right)$$

Notice that τ_d is a non-dimensional quantity that expresses the delay t_d between the activation of Y and that of Z relative to the characteristic lifetime of Y.

Derivation of $\overline{\theta}_{YZ}$

In this section we evaluate the fraction of time spent by Y over the threshold K_{yz} during one cycle. In order to do this we first evaluate the time spent by Y over K_{yz} when increasing, then the time spent by Y over K_{yz} when decreasing. The sum of these provides the actual time spent by Y activating Z and the fraction of time in one cycle of oscillation is obtained by simply dividing by the period of oscillation T.

Under the action of X, Y increases for a time $T\overline{\theta}_{XY}$. The actual time spent by Y over K_{yz} in the increasing branch, remembering that $\alpha_y T = \tau$, is given by

$$T\overline{\theta}_{XY} - t_d = T\overline{\theta}_{XY} - \frac{T}{\tau}\left[\log\left(\frac{\beta_y^{\text{on}} - \beta_y^{\text{off}}}{\beta_y^{\text{on}} - \alpha_y K_{yz}}\right) + \log\left(\frac{1 - e^{-\tau(1-\overline{\theta}_{XY})}}{1 - e^{-\tau}}\right)\right]$$

When X stops activating Y, Y decays according to the formula

$$Y(t) = \frac{\beta_y^{\text{off}}}{\alpha_y} - \left(\frac{\beta_y^{\text{off}}}{\alpha_y} - Y_{\max}\right) e^{-\alpha_y t}$$

so that the time spent above K_{yz} when Y is decreasing can be evaluated by finding the value t for which:

$$K_{yz} = \frac{\beta_y^{\text{off}}}{\alpha_y} - \left(\frac{\beta_y^{\text{off}}}{\alpha_y} - Y_{\max}\right) e^{-\alpha_y t}$$

rearranging the terms one gets:

$$e^{-\alpha_y t} = \frac{\alpha_y K_{yz} - \beta_y^{\text{off}}}{\alpha_y Y_{\max} - \beta_y^{\text{off}}}$$

Passing to the logarithm and remembering that $\alpha_y T = \tau$:

$$t = \frac{T}{\tau}\log\left(\alpha_y Y_{\max} - \beta_y^{\text{off}}\right) - \frac{T}{\tau}\log\left(\alpha_y K_{yz} - \beta_y^{\text{off}}\right)$$

Using the expression for $Y_{\max}$ then $\beta_y^{\text{off}} - \alpha_y Y_{\max} = \frac{(1-e^{(-\tau\overline{\theta}_{XY})})(\beta_y^{\text{on}} - \beta_y^{\text{off}})}{1-e^{-\tau}}$ and so the time spent over the threshold when Y is decreasing is:

$$\frac{T}{\tau}\left[\log\left(\frac{\beta_y^{\text{on}} - \beta_y^{\text{off}}}{\alpha_y K_{yz} - \beta_y^{\text{off}}}\right) + \log\left(\frac{1 - e^{-\tau\overline{\theta}_{XY}}}{1 - e^{-\tau}}\right)\right]$$

Adding up the time spent by Y over K_{yz} in the increasing branch and in the decreasing one we obtain:

$$\begin{aligned} T\overline{\theta}_{YZ} =& T\overline{\theta}_{XY} + \frac{T}{\tau}\left[\log\left(\frac{\beta_y^{\text{on}} - \beta_y^{\text{off}}}{\alpha_y K_{yz} - \beta_y^{\text{off}}}\right) + \log\left(\frac{1 - e^{-\tau\overline{\theta}_{XY}}}{1 - e^{-\tau}}\right) + \right. \\ =& \left. - \log\left(\frac{\beta_y^{\text{on}} - \beta_y^{\text{off}}}{\beta_y^{\text{on}} - \alpha_y K_{yz}}\right) - \log\left(\frac{1 - e^{-\tau(1-\overline{\theta}_{XY})}}{1 - e^{-\tau}}\right)\right] = \\ =& T\overline{\theta}_{XY} + \frac{T}{\tau}\left[\log\left(\frac{1 - e^{-\tau\overline{\theta}_{XY}}}{1 - e^{-\tau(1-\overline{\theta}_{XY})}}\right) + \log\left(\frac{\beta_y^{\text{on}} - \alpha_y K_{yz}}{\alpha_y K_{yz} - \beta_y^{\text{off}}}\right)\right] \end{aligned}$$

and by dividing by the period T one finds the fraction of time spent by Y over the threshold K_{yz} in each cycle:

$$\overline{\theta}_{YZ} = \overline{\theta}_{XY} + \frac{1}{\tau}\left[\log\left(\frac{1 - e^{-\tau\overline{\theta}_{XY}}}{1 - e^{-\tau(1-\overline{\theta}_{XY})}}\right) + \log\left(\frac{\beta_y^{\text{on}} - \alpha_y K_{yz}}{\alpha_y K_{yz} - \beta_y^{\text{off}}}\right)\right]$$

Network Motifs with OR gate

In the main text we focussed our attention on network motifs with AND gates. In this section we discuss the case of FFLs with an OR gate, often arising in real biological networks. We use the CFFL-1 and IFFL-1 as prototypes to investigate the response to the frequency and temporal profile (shape) of oscillating transcription factors. As for the AND case, in the OR case our simulations show that the general behavior remains qualitatively the same for all other types of FFLs.

CFFL-1 with OR gate

Contrary to the case of an AND gate, the expression of a CFFL-1 with OR gate is started immediately following the stimulation by a transcription factor X. Due to the accumulation of Y when the signal X is removed the expression of Z does not stop immediately but is delayed until Y falls below the threshold K_{yz} (Figure S2). Such FFL protects the expression of Z from transient loss of the transcriptional activity of X [2, Chap. 4].

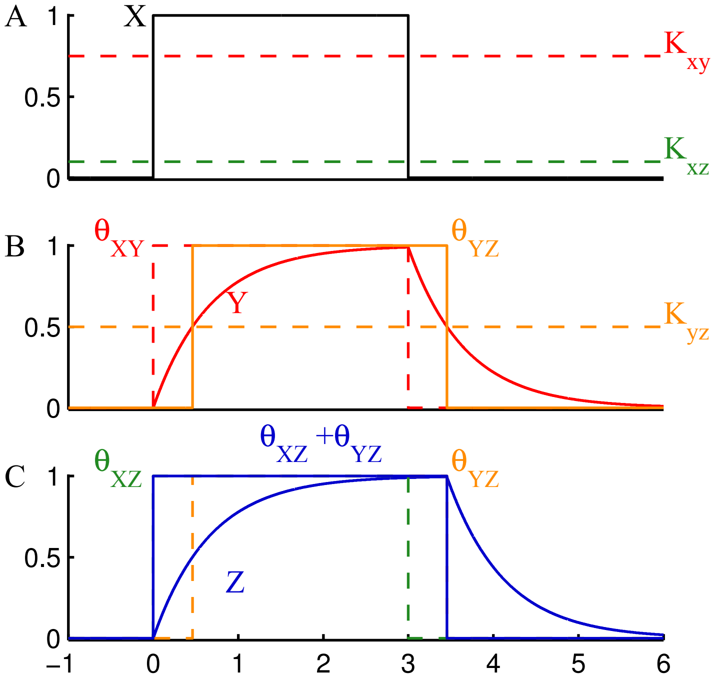

Figure S2. Response of CFFL-1 with OR gate to a step-like stimulation. The figure shows the typical response of a CFFL-1 with OR gate to a transient transcription factor X. A step-like transcription factor X activates simultaneously both branches of the FFL **(A)**. Under the stimulus X (signal θ_{XY}), Y starts accumulating **(B)**, and the transcription of Z is activated because the signal $\theta_{XZ} + \theta_{YZ}$ immediately promotes its expression (C). When X is removed Y starts degrading (B) but the expression of Z goes on until Y falls below the threshold K_{yz}. The following parameters have been used: $\beta_y^{\text{on}} = \beta_z^{\text{on}} = \alpha_y = \alpha_z = 1.5$, $\beta_y^{\text{off}} = \beta_y^{\text{off}} = 0$, $K_{xy} = 0.75$, $K_{xz} = 0.1$, $K_{yz} = 0.5$.

When stimulated with an oscillatory transcription factor the average value of expression of Z for different periods of oscillation shows patterns similar to those already seen for the CFFL-1 with AND gate. The differences between the two cases arise because while in the case of AND one of the two branches acts as a brake on the expression of the gene, in the OR case the two branches both act independently and cooperatively to the final response enhancing the overall expression of the gene with respect to the corresponding cases of CFFL-1 with AND gate.

The four classes of responses for the CFFL-1 with OR gate are shown in Figure S3. The results can be explained as follows:

A) $K_{xy} < K_{xz}$, $K_{yz} < \overline{Y}$ the average expression of the gene increases with the frequency of oscillation. Since $K_{yz} < \overline{Y}$ the duration of the signal θ_{XY} increases as the frequency of oscillation increases, till eventually Y is always above the threshold K_{yz} and the gene is fully expressed. In this case the signal θ_{XZ} is contained in the signal θ_{XY} so that the relative delays between the activation of the branches does not influence the final outcome of expression. This is the opposite of what happens in the corresponding case of a CFFL-1 with AND gate in which the XZ branch was actually the master regulator of the expression of Z keeping its average value constant to a relatively low value;

B) $K_{xy} < K_{xz}$, $K_{yz} > \overline{Y}$ the average value of Z diminishes as the frequency of oscillation increases, due to the fact that since $K_{yz} > \overline{Y}$ the duration of the YZ signal decreases. The relative delays between the θ_{XY} and θ_{XZ} affects the average response. In fact, since $K_{yz} > \overline{Y}$ the delay between the signal θ_{XY} and θ_{YZ} is relatively high. For the left signal for which the delay between θ_{XY} and θ_{XZ} is relatively short this means that the two signals θ_{YZ},θ_{XZ} do not overlap much and the expression is kept high. In the case of the symmetric and the right signals as the frequency increases the overlap increases (due to the increasing delay between θ_{XY} and θ_{YZ}) and the duration of θ_{YZ} decreases causing the gene expression to be relatively low even at low frequencies. The major difference with the corresponding case of CFFL-1 with AND gate is that the expression is not completely turned off because X keeps promoting Z through the XZ branch even at high frequencies;

C) $K_{xy} > K_{xz}$, $K_{yz} < \overline{Y}$ the gene expression is fully switched on as the frequency of oscillation of X increases because the duration of the signal θ_{YZ} increases with the frequency. Since K_{xz} is low the activity of X through the XZ branch is always quite high and the gene expression is always relatively high. Signals with short delay between θ_{XY} and θ_{XZ} show a sharp transition because the θ_{YZ} signal has a short delay with respect to θ_{XY} making the θ_{YZ} overlap with θ_{XZ} for broader ranges of frequencies. The case differs from the corresponding one with AND gate because in that case the XY branch has a limiting effect on the expression of the gene, causing the average value of Z to be low for low frequencies, and being higher but limited (by the θ_{XZ} signal) for high frequencies;

D) $K_{xy} > K_{xz}$, $K_{yz} > \overline{Y}$ In this case the effects of X through the XZ branch always mask the contribution to the expression of Z by YZ for low frequencies, at higher frequencies the XZ branch governs the expression of Z alone. The final result is that the average value of Z is constant at different frequencies and it is insensitive to the temporal profile of X. The difference with respect to the AND gate is that there the expression of Z is limited by the short duration of the YZ signal at low frequencies and it is switched off at high frequencies because of the reduced value of $\overline{\theta}_{YZ}$.

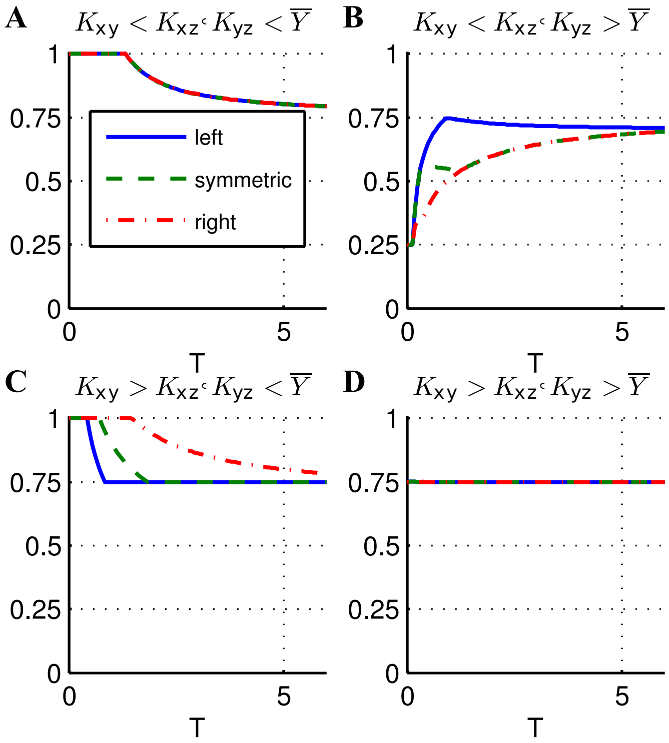

Figure S3. CFFL-1 OR gate, average response of the gene Z at stationary regime for various configurations of the parameters. For all the plots the values $\beta_y^{\mathrm{on}} = \beta_z^{\mathrm{on}} = \alpha_y = \alpha_z = 4.15$ have been used. The thresholds of activation for the various cases are: **(A)** $K_{xy} = 0.1 < K_{xz} = 0.75$, $K_{yz} = 0.25 < \overline{Y} = 0.75$; **(B)** $K_{xy} = 0.1 < K_{xz} = 0.75$, $K_{yz} = 0.8 > \overline{Y} = 0.75$; **(C)** $K_{xy} = 0.75 > K_{xz} = 0.1$, $K_{yz} = 0.125 < \overline{Y} = 0.25$; **(D)** $K_{xy} = 0.75 > K_{xz} = 0.1$, $K_{yz} = 0.6 > \overline{Y} = 0.25$.

IFFL-1 with OR gate

A gene Z regulated by an IFFL-1 with OR gate is expressed in the cell when no transcription factor X is present. When X stimulates the FFL, although an inhibitor Y is promoted no variations of the value of Z is detected, because the presence of X ensures that the gene is still transcribed. Only when X is removed, the presence of the inhibitor Y causes a temporal decrease of Z, whose expression is promptly restored when Y falls below the threshold of inhibition (Figure S4).

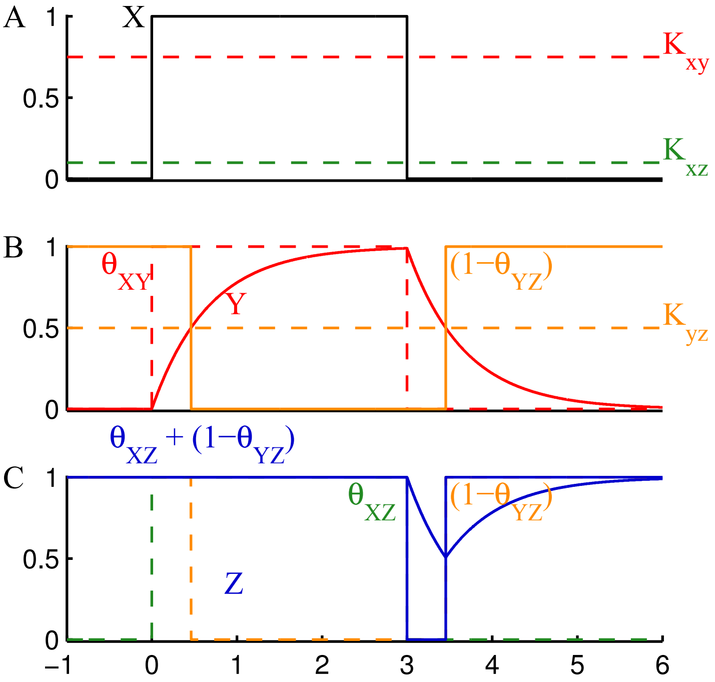

Figure S4. Response of IFFL-1 with OR gate to step-like stimulation. The figure shows the typical response of an IFFL-1 with OR gate to a transient transcription factor X. When X is not present Z is expressed in the absence of Y. When a transient activation of the IFFL-1 is stimulated by a transient transcription Z is not modified because even when Y is over the threshold value K_{yz}, the presence of X keeps promoting its expression. When X is removed the expression of Z is temporarily suspended because $\theta_{XZ} = 0$ and $\theta_{YZ} = 1$ so that the final signal $\theta_{XZ} + (1 - \theta_{YZ})$ acting on Z is 0. After a certain delay Y falls below the threshold of inhibition K_{yz} and Z activity is restored. The following parameters have been used: $\beta_y^{\rm on} = \beta_z^{\rm on} = \alpha_y = \alpha_z = 1.5$, $\beta_y^{\rm off} = \beta_y^{\rm off} = 0$, $K_{xy} = 0.75$, $K_{xz} = 0.1$, $K_{yz} = 0.5$.

In the case of stimulation with an oscillating transcription factor the average value of Z varies with the frequency as shown in Figure S5. The various cases are explained as follows:

A) $K_{xy} < K_{xz}$, $K_{yz} < \overline{Y}$ At high frequencies Y is always above the threshold of inhibition causing the gene Z to be expressed only upon the stimulation of X through the XZ branch. At low frequency Y spends less and less time above the threshold of inhibition causing an increase in the average

value of Z. This case is different from the AND gate case because there the gene would never have responded to the stimulation by the transcription factor;

B) $K_{xy} < K_{xz}$, $K_{yz} > \overline{Y}$ At high frequencies Y does not reach the threshold of inhibition K_{yz} causing Z to be fully expressed. For lower frequencies it spends some time above the threshold of inhibition causing Z to be expressed at lower values. The temporal profile of the signals influences the sharpness of the transition. For signals having short delays between θ_{XY} and θ_{XZ} the region in which X is active and Y is not inhibiting Z overlap for a broader region of frequencies determining the transition to appear more sharply;

C) $K_{xy} > K_{xz}$, $K_{yz} < \overline{Y}$ At low frequencies of oscillation Y inhibits Z only when X is present so that Z is always expressed at high concentrations. As the frequency increases it always exerts its inhibitory effect, so that the average level of expression drops as it would be only regulated by X. The relative delay of activation between θ_{XY} and θ_{XZ} determines the time of overlap between X and Y providing responses of different sharpness. Due to the OR gate makes the gene expression does not decrease as in the corresponding case of IFFL-1 with AND gate;

D) $K_{xy} > K_{xz}$, $K_{yz} > \overline{Y}$ with this set of parameters at low frequencies Y inhibits Z only when X is present so that Z is always fully expressed. At high frequencies Y is always below the threshold of inhibition, and as a result the gene is always fully expressed and its value is not affected by the period of oscillation. This differs from the AND case for which the gene is never fully activated and its average value increases with the frequency of oscillation.

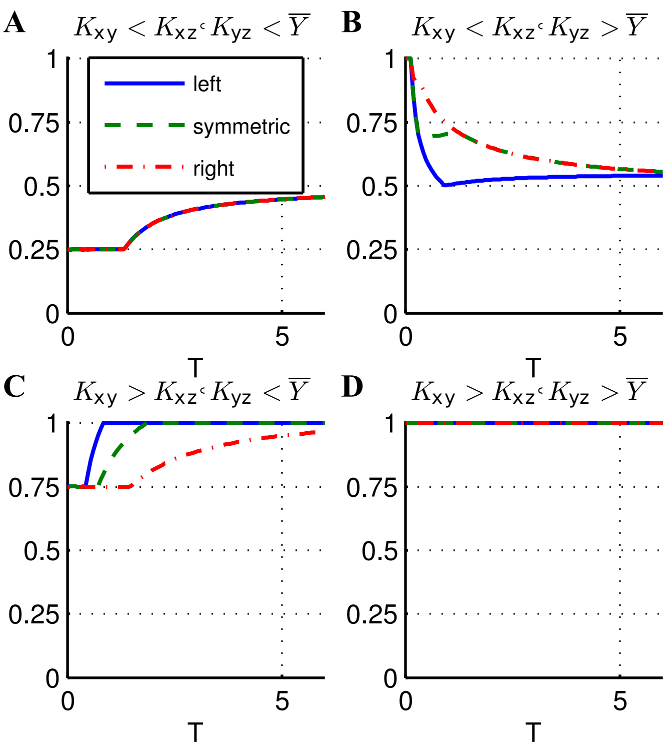

Figure S5. IFFL-1 with OR gate, average response of the gene Z at stationary regime. For all the plots the values $\beta_y^{\text{on}} = \beta_z^{\text{on}} = \alpha_y = \alpha_z = 4.15$ have been used. The thresholds of activation for the various cases are: **(A)** $K_{xy} = 0.1 < K_{xz} = 0.75$, $K_{yz} = 0.25 < \overline{Y} = 0.75$; **(B)** $K_{xy} = 0.1 < K_{xz} = 0.75$, $K_{yz} = 0.8 > \overline{Y} = 0.75$; **(C)** $K_{xy} = 0.75 > K_{xz} = 0.1$, $K_{yz} = 0.125 < \overline{Y} = 0.25$; **(D)** $K_{xy} = 0.75 > K_{xz} = 0.1$, $K_{yz} = 0.6 > \overline{Y} = 0.25$.
